# Effect of Ageing on Systemic Inflammatory Response in Acute Pancreatitis

**DOI:** 10.1155/2012/270319

**Published:** 2012-01-15

**Authors:** Marcel Cerqueira Cesar Machado, Ana Maria Mendonça Coelho, Luiz Augusto Carneiro D'Albuquerque, Sonia Jancar

**Affiliations:** ^1^LIM-37, Department of Gastroenterology, University of Sao Paulo Medical School, 01246-903 Sao Paulo, SP, Brazil; ^2^Department of Immunology, Institute for Biomedical Sciences, University of Sao Paulo, 05508-900 Sao Paulo, SP, Brazil

## Abstract

Elderly patients show increased incidence of multiple organ dysfunction in acute pancreatitis possibly due to bacterial translocation. This is associated with increased susceptibility to infections in older people. Several reports have related this increased susceptibility to a proinflammatory status called *inflammaging*, which decreases the capacity of the immunological system to respond to antigens. Cellular senescence also contributes to this low-grade chronic inflammation in older subjects. We discuss here the effect of ageing on systemic inflammation, focusing on that induced by acute pancreatitis and some of the mechanisms involved. It is important to understand the immunological changes in the elderly to adjust treatment strategies in order to reduce the morbidity and mortality associated with acute pancreatitis and other conditions that lead to systemic inflammation.

## 1. Introduction

Several reports investigating ageing physiopathology have stated that old age is followed by a low-grade inflammatory process [1], which may be upregulated during sepsis, surgical procedures, and ischaemic/reperfusion injury. This low-grade inflammatory state is called *inflammaging, *a concept supported by several reports [2–4]. This is probably related to antigenic stress throughout life that may have caused exhaustion of immunological cells, thus decreasing the capacity of the immunological system to respond to antigens [5]. A concept that has been established is one of senescent cells being able to secrete many factors including growth factors, proteases, and cytokines that can induce inflammation [6]. Cellular senescence is a situation where the cell has an irreversible proliferative arrest due to potential oncogenic stress. This cellular state, limited to proliferative cells, has been recognized as a tumour suppressive mechanism. Senescent cells remain metabolically active, producing an array of tumour-suppressing and proinflammatory substances. Senescent cells probably contribute to the chronic inflammatory state related to ageing. This subject was extensively discussed in two important reviews, which also analyzed the molecular mechanisms and cellular pathways associated with cellular senescence [6, 7].

 There is also clear evidence that elderly patients are more susceptible to infections after surgical procedures than young patients, and this could be related to the pro-inflammatory status of the elderly patients. This increased susceptibility to infections could be the reason for the increased postoperative morbidity and mortality observed in elderly patients [8]. However, there are reports showing that selection and appropriate care make the outcome of even major surgical procedures in elderly patients similar to that in young patients [9–15]. Thus, it seems that multiple factors influence the postoperative outcome of ageing people, including comorbidities and immunological status. It is therefore important to understand the immunological characteristic changes in the elderly to adjust treatment strategies in order to reduce the morbidity and mortality associated with surgery, infection, and acute pancreatitis (AP) among other conditions that induce systemic inflammation.

## 2. Molecular Mechanisms of Age-Related Inflammation

The molecular mechanisms involved in the chronic low-grade inflammation of elderly people are poorly understood. Up- or downregulation of genes related to the development of inflammation may be involved in the process. A recent report pointed to the potential role of the poly(ADP-ribose) polymerase-1 gene in inflammation and the ageing process [16]. It has also been found that aged animals with sepsis present higher splenic tissue concentration of alpha-2A adrenergic receptors and phosphodiesterases (related to the autonomic nervous system) and increased CD14 and toll-like receptor-4 expression (related to innate immunity) when compared to young animals. The authors of this report concluded that hyperinflammation in older animals is related not only to the innate immune response but also to the upregulation of the adrenergic autonomic nervous system that may contribute to increased proinflammatory cytokine production [17].

 The heat shock protein 70 has been shown to modulate the systemic response of an aged host to sepsis, preventing gut cell apoptosis and systemic inflammation [18]. In a study of partial liver ischaemia/reperfusion injury, the recruited neutrophils from aged rats were observed to have a higher-activation state when compared to those from young animals and that the hepatic heat shock protein 70 was less expressed in old animals [19]. It is possible to speculate that the diminished expression of heat shock protein 70 in the elderly population may be responsible for the higher-bacterial translocation and organ dysfunction observed in elderly patients with AP [20].

## 3. Systemic Inflammation and Ageing

Systemic inflammation such as that induced by sepsis is accompanied by a burst in the production of proinflammatory cytokines, whose serum levels are higher in elderly patients compared to the young [21].

Elderly patients usually develop an exaggerated inflammatory response after surgery and this has been attributed to the proinflammatory status of older people [22]. However, when pro-inflammatory cytokine levels are analyzed, contradictory reports are found in the literature: (a) increased and delayed levels of interleukin (IL)-6 have been observed in elderly patients after surgery [23]; (b) monocyte activation and hypercytokinemia have been observed in elderly patients after surgical intervention [24]; (c) significantly higher levels of tumour necrosis factor (TNF)-alpha, IL-6, and IL-1-beta are produced by mitogen-stimulated peripheral mononuclear cells from the elderly when compared to young subjects [19]. Contrarily, blood monocytes from elderly patients with pneumonia produce less proinflammatory cytokines (IL-1beta, TNF-alpha, and IL-8) upon stimulation than those from young patients [25]. The controversy is further increased by reports showing that young and old patients with community-acquired pneumonia [26] have similar serum levels of IL-6 and IL-10, and that there is no difference in serum pro-inflammatory cytokine levels between elderly and young patients after coronary surgery.

Ischaemia/reperfusion is another condition that induces systemic inflammation. Liver ischaemic/reperfusion injury is increased in older animals due to an exaggerated production of TNF-alpha. Inhibition of TNF-alpha production reverses the effect of ageing in ischaemic/reperfusion injury [27]. Ischaemic preconditioning protects young livers from ischaemic/reperfusion injury; however, this strategy increases liver damage in old livers [27]. This information is extremely important for liver surgeons dealing with hepatic ischaemia.

It is important to consider that during systemic inflammation, organ-specific alterations may take place and contribute to systemic inflammation. The lungs are particularly affected, which release a second wave of mediators that may potentiate systemic inflammation. In an experimental model of systemic inflammation, it was found that lung injury was much more intense in aged compared to young mice, as demonstrated by increased inducible nitric oxide synthase expression and decreased extracellular superoxide dismutase levels [28].

## 4. Acute Pancreatitis and Ageing

Severe AP is associated with high morbidity and mortality due to local pancreatic complications and multiple organ dysfunction [29, 30]. Although the mortality rate of severe AP has decreased in recent years, it is still around 20 to 25% [31, 32].

 Advanced age has been considered an independent prognostic factor for mortality in AP [33]. The presence of complications such as infected pancreatic necrosis in the aged population is associated with a mortality rate of up to 50% [34]. In elderly patients with AP, in spite of similar occurrence of local complications, a substantial increase in multiple organ failure has been found [20]. However, it has not been clearly demonstrated that elderly patients with AP have different outcomes when compared to younger patients [35, 36]. Despite the severity of local inflammation being higher in younger compared to older patients, the score that measures the clinical severity of the disease (APACHE II, acute physiology, and chronic health evaluation) is higher in elderly patients [35]. To our understanding, this indicates that the severity of systemic response in elderly patients is more intense.

 Although ageing is related to increased mortality due to organ failure in AP, no differences in local complications between young and elderly patients have been observed [37, 38]. Indeed, advanced age and associated diseases do not influence the occurrence of pancreatic necrosis [39]. The factors related to the increased incidence of organ failure in elderly patients, however, are not well understood and may be related to changes in the innate immune system or to an abnormal elevation of proinflammatory cytokines in the bowel, which facilitates bacterial translocation. In a recent study, we found that 2-year-old rats with taurocholic acid-induced AP presented much higher-bacterial translocation than the young ones (in preparation). The available information on the effect of ageing on AP inflammation is summarized in Table 1.

## 5. Future Studies and Conclusions

Studies addressing intestinal alterations during AP in older animals should help to elucidate the mechanisms involved in bacterial translocation that are related to multiple organ failure in AP.

 New strategies that reduce the exaggerated inflammatory response in elderly patients are needed to decrease morbidity and mortality in elderly patients with AP. Our group has shown some strategies that reduce the inflammatory response in experimental AP, such as lavage of the peritoneal cavity [40], treatment with hypertonic saline solution [41, 42], treatment with platelet-activating factor antagonists [43], and pentoxifylline [44]. It would be desirable to investigate if these strategies are also effective in reducing the exaggerated inflammatory response in older animals with AP.

Specialized diets have been proposed to increase innate immunity and protect elderly patients from infections and should also be tested in elderly AP patients [45].

 In conclusion, advanced age is considered an independent prognostic factor for mortality in acute pancreatitis, but the mechanisms involved in this process are not completely understood. Organ failure seems to be the most important factor responsible for the higher mortality rate observed in elderly patients since local complications are similar in young and older patients.

 It is conceivable that different therapeutic strategies should be employed in young and old people to control AP, and clinical trials on anti-inflammatory and antimicrobial drugs in AP should consider patient age as an important issue.

## Figures and Tables

**Table 1:** Effect of ageing on acute pancreatic inflammation.

	Young	Elderly	references
Mortality	↑	↑↑	[33]
Organ failure	↑	↑↑	[20]
Local complications	↑	↑	[37, 38]
Bacterial translocation	↑	↑↑	*
Systemic inflammation	↑	↑↑	[35]

*Coelho AMM, Machado MCC, Sampietre SN et al. Aging is related to increased intestinal damage and bacterial translocation in acute pancreatitis in rats (in preparation).
